# Non-Alcoholic Fatty Liver Disease among Type-2 Diabetes Mellitus Patients in Abha City, South Western Saudi Arabia

**DOI:** 10.3390/ijerph15112521

**Published:** 2018-11-11

**Authors:** Abdullah A. Alsabaani, Ahmed A. Mahfouz, Nabil J. Awadalla, Mustafa Jafar Musa, Suliman M. Al Humayed

**Affiliations:** 1 Department of Family and Community Medicine, College of Medicine, King Khalid University, P.O. Box 641, Abha 61421, Saudi Arabia; dr.alsabaani@hotmail.com (A.A.A.); njgirgis@yahoo.co.uk (N.J.A.); 2 Department of Epidemiology, High Institute of Public Health, Alexandria University, Alexandria 21511, Egypt; 3 Department of Community Medicine, College of Medicine Mansoura University, Mansoura 35516, Egypt; 4 Department of Radiology, College of Applied Medical Sciences, King Khalid University, P.O. Box 641, Abha 61421, Saudi Arabia; mustafamusa363@yahoo.com; 5 Department of Internal Medicine, College of Medicine, King Khalid University, P.O. Box 641, Abha 61421, Saudi Arabia; s_humayed@yahoo.com

**Keywords:** non-alcoholic fatty liver disease, type-2 diabetes mellitus, Saudi Arabia

## Abstract

The objective of this study was to determine the prevalence and the factors associated with non-alcoholic fatty liver disease (NAFLD) among type-2 diabetes mellitus (T2DM) patients in Abha City, Southwestern Saudi Arabia. Using a cross-sectional study design, a representative sample of 245 T2DM patients were recruited from all primary healthcare centers in Abha city. A detailed medical history as well as laboratory investigations were done. NAFLD was diagnosed using abdominal ultrasound examination. The overall prevalence of NAFLD was 72.8% (95% CI: 66.6%–78.1%). In a multivariable regression analysis, the risk of NAFLD was significantly higher among overweight T2DM patients (aOR = 6.112, 95% CI: 1.529–4.432), Obese (aOR = 10.455, 95% CI: 2.645–41.326), with high ALT of more than 12 IU/L (aOR = 2.335, 95% CI: 1.096–5.062), moderate diet-compliant patients (aOR = 2.413, 95% CI: 1.003–5.805) and poor diet-compliant patients (aOR = 6.562, 95% CI: 2.056–20.967). On the other hand, high HDL (high density cholesterol) (in mg/dL) was a protective factor for NAFLD (aOR = 0.044, 95% CI: 0.005–0.365). It was concluded that NAFLD is a common association of T2DM. Increasing BMI (Body mass index), lower HDL level, and poor dietary control are significant factors associated with NAFLD among T2DM patients. Health education to improve dietary control and avoid excessive weight gain, testing for NAFLD among diabetic patients, especially those with abnormal BMI and HDL, are recommended for early detection and to ensure optimal levels of HDL.

## 1. Introduction

Non-alcoholic fatty liver disease (NAFLD), defined as the presence of hepatic steatosis in the absence of secondary causes, is emerging as a public health issue worldwide, with a global pooled prevalence, by imaging, of 25.24% among the general population [1]. NAFLD is the most common chronic liver disease in the industrialized countries; also, it is the major cause of cryptogenic cirrhosis. NAFLD is a disease that can occur in all sexes, ages, and ethnic groups. The major risk factors of NAFLD are obesity, hyperlipidemia, diabetes mellitus, and metabolic syndrome (insulin resistance syndrome). The latter represents the strongest risk factor [2]. Due to epidemics of DM (diabetes mellitus) and Obesity in industrialized countries, this will also lead to a dramatic rise in the prevalence of NAFLD. There is a very high rate of NAFLD in patients with T2DM [3].

The association between NAFLD and type-2 diabetes mellitus (T2DM) has been well established, and can be explained by the insulin-resistance and compensatory hyper-insulinemia progressing to defective lipid metabolism and hepatic triglyceride (TG) accumulation in NAFLD or to b-cell dysfunction in T2DM [4]. Evidence showed that NAFLD-T2DM association is bidirectional [5].

In the Kingdom of Saudi Arabia, in a one center study in 2012, a prevalence of NAFLD of 16.6% was documented among the general population. NAFLD, as detected by ultrasound, was common in Saudi patients with type-2 diabetes [6]. The actual prevalence of NAFLD among diabetic patients is usually underestimated in ultrasonongrahic studies as compared with studies that rely on the histological diagnosis (liver biopsy); however, ultrasonography is safe and cost effective for screening a large number of patients, whereas liver biopsy is limited by cost and associated morbidities [2,7]. 

Data regarding NAFLD among T2DM in Abha city are lacking. The main objectives of this study were to determine the prevalence of NAFLD among patients with type-2 diabetes in Abha City, southwestern Saudi Arabia, and to determine the most important associated factors.

## 2. Materials and Methods

### 2.1. Study Design and Settings

The study was cross-sectional, targeting patients attending primary health care Diabetes clinics at Abha city. The main inclusion criteria were adult patients (18 years and above) with T2DM, while patients with coexisting liver disease and those who consume alcohol or take steatogenic drugs were excluded from the study.

### 2.2. Sample Size and Sampling

Using sampling formula for a single cross-sectional survey, a minimal sample size of 267 participants was calculated, based on 95% confidence interval and absolute precision of 6%. Since there is no prior estimate for the prevalence of NAFLD among T2DM population in Abha City, an estimate of 48% derived from a study conducted in the Jazan region of Saudi Arabia [8] was utilized. To account for loss of cases, it was decided to include 345 subjects in the study.

Patients were selected from the 7 Abha City primary health care centers using a systematic random sampling method to ensure a high degree of randomization.

### 2.3. Socio-Demographic, Clinical and Laboratory Data

Patient files were revised to collect data regarding age, gender, duration of diabetes, drug history, medical history, diabetes management compliance (follow up, diet, drug intake, and exercise). The following laboratory investigations were done; HbA1c, cholesterol, triglycerides, high density cholesterol (HDL), low density cholesterol (LDL), alanine aminotransferase (ALT), and aspartate aminotransferase (AST).

Weight and standing height were measured. Body mass index (BMI) was calculated as weight (kg)/height (m^2^). Patients with BMIs of less than 25 were classified as normal, over 25 classified as overweight, and over 30 were classified as obese.

### 2.4. Ultrasound Examination

The patients were asked to fast for 6 to 8 h before the abdominal ultra sound examination. The examination was taken with the patient supine, and ultrasound gel was applied to the abdomen. Longitudinal and transverse scanning was performed. The texture and measurement of the liver were recorded. The examination was performed by a single, expert radiologist using a sensitive ultrasound machine with a 3.5 MHZ convex transducer. The normal liver parenchyma has homogenous echo texture, with echogenicity equal or slightly greater than that of the renal cortex and spleen. The fatty liver showed higher echogenicity than the renal cortex in both side. Different grade of steatosis have been proposed based on the intensity of the echogenicity, provided that the gain setting is optimum. When the echogenicity was increased, this was considered to be grade 1. When the branches of the portal vein were obscured, it was grade 2, and when the echogenicity of the liver covered the diaphragmatic outline, it was classified as grade 3.

### 2.5. Statistical Analysis

Arithmetic mean, standard deviation, and proportions (with 95% confidence intervals) were used to present the data. Crude odds ratios (cOR) and 95% confidence intervals were calculated for univariate analysis. The logistic regression model was used to evaluate factors associated with NAFLD. Adjusted odds ratios (aOR) and their 95% confidence intervals were calculated. Variables included in the model were gender, age, BMI, history of hypertension, HbA1c, cholesterol, triglycerides, HDL, LDL, ALT, AST, duration of T2DM and patients management compliance (follow up, diet, drug, and exercise).

### 2.6. Ethical Approval

The research was approved by the Research Ethics Committee, College of Medicine, King Khalid University (HA-O7-B-012, dated June 2016). Written informed consents were collected from all patients enrolled in the study. Permission was also obtained from the Aseer Directorate of Health.

## 3. Results

The present study included 245 T2DM patients. They included 162 (66.1%) males and 83 (33.9%) females. Their age ranged from 20 to 100 years, with an average of 57.1 ± 13.5 and a median of 51. The majority were married (199, 81.2%) and school (primary, intermediate or secondary) educated (133, 54.2%). Current smokers represented 17.6% (43) of the study sample. The duration of T2DM ranged from 1 to 42 years, with an average of 10.1 ± 8.3 and a median of 8 years. The majority were using only oral hypoglycemic (146, 59.6%). The rest were treated by insulin alone (32, 13.1%) or combined Insulin and oral hypoglycemic (67, 27.3%). Regarding co morbidities, 46.1% (113) were hypertensive and 6.5% (16) had ischemic heart diseases. The glycosylated hemoglobin (HbA1c %) ranged from 5.7% to 14%, with an average of 8.7% ± 2.1% and a median of 8.2%. Only 29 (11.8%) T2DM patients had a value of less than 6.5%; the rest were above.

The present study showed that NAFLD was found among 178 T2DM patients, giving a prevalence of 72.7% (95% CI: 66.6%–78.1%). Two-thirds of NAFLD patients were of grade 1 (121, 68%); the rest were grade 2 (50, 28.1%) and grade 3 (7, 3.9%). The vast majority of cases were of the diffuse type (169, 94.9), and very few were of the focal type (9, 5.1%).

Table 1, Table 2 and Table 3 show the risk factors associated with NAFLD among T2DM patients. Table 1 shows that in a multivariable regression analysis for the personal and clinical factors associated with NAFLD among the study sample, only BMI was found to be significant factor. Over weight (BMI of 25–29.9) T2DM patients had 6 times the risk (aOR = 6.112, 95% CI: 1.529–4.432) of NAFLD compared to normal-weight patients. Similarly, Obese (BMI of ≥30) T2DM patients had 10 times the risk (aOR = 10.455, 95% CI: 2.645–41.326) of NAFLD compared to normal-weight patients.

Table 2 shows the biochemical factors associated with NAFLD among the study sample. T2DM patients with high ALT (>12 IU/L) had significantly more than two times the risk (aOR = 2.335, 95% CI: 1.096–5.062) of NAFLD compared to T2DM patients with normal ALT (<12 IU/L). On the other hand, the study showed that high HDL (in mg/dL) was a protective factor for NAFLD (aOR = 0.044, 95% CI: 0.005–0.365).

Table 3 shows the compliance factors associated with NAFLD among the study sample of T2DM patients. Moderately diet-compliant T2DM patients had 2 times the risk (aOR = 2.413, 95% CI: 1.003–5.805) of NAFLD compared to adequately diet-compliant patients. Similarly, poor diet-compliant T2DM patients had 6 times the risk (aOR = 6.562, 95% CI: 2.056–20.967) of NAFLD compared to adequately diet-compliant patients.

## 4. Discussion

The present study found a prevalence of NAFLD among T2DM patients of 72.7% (95% CI: 66.6%–78.1%). Almost similar figures were found among T2DM patients in India (56.5%) [9], Italy (59.6%) [4], Sri Lanka (62.6%) [10] and Nigeria (68.8%) [11]. In a recent meta analysis study including 24 articles [12], the prevalence ranged from 29.6% to 87.1%, with a pooled prevalence of 59.7% (95% CI: 54.3%–64.9%). In Saudi Arabia, a cross sectional study in Jazan region [8] found a prevalence of 47.8% (95% CI 41.1–54.6).

The results of the present study revealed that the increase in patients’ BMI was a significant independent factor associated with higher risk of NAFLD among studied T2DM patients. Similar association has been reported in previous studies [10,12,13]. Whether this relationship is just an association or it has any causative effect on NAFLD could not be determined by the current study. However, the positive dose response relationship between BMI and the risk of NAFLD detected by the present study may increases the strength of this association. Taking into consideration the higher risk of NAFLD among obese and overweight T2DM patients, as well as non-diabetic individuals [14], weight loss has been recommended among overweight and obese individuals by previous studies [15,16,17].

Our study revealed that elevated ALT level was also an independent factor associated with higher risk of NAFLD. Similar finding has been observed by previous studies [18,19,20]. ALT level is raised in up to 80% of NAFLD patients, and is an important biomarker for hepatocellular damage [21]. However, our study failed to find a significant association between higher level of AST and NAFLD. Recent studies suggested that AST levels do not correlate well with degree of hepatic steatosis, and may be normal in high grades of steatosis [15,22].

The finding of the current study suggests that low serum level of HDL was a significant independent predictor of NAFLD. On the other hand, higher serum triglyceride and LDL were less associated with NAFLD. The same results were observed in a previous Polish study [23]. Therefore, it seems important in prevention of NAFLD among diabetic patients to reach the appropriate level of serum HDL [23].

The results of the present study revealed that moderate and poor diet-compliant T2DM patients had a higher risk of NAFLD compared to adequately diet-compliant patients. A high calorie diet, the abundance of saturated fats and refined carbohydrates, the intake of sugar-sweetened beverages, and a high fructose intake were found to be associated with a greater risk of NAFLD [19,24,25]. It seems that poor dietary compliance and insulin resistance in T2DM have a synergistic effect on the development of NAFLD [26].

The results of the current study suggest that HbA1c levels are not a suitable predictor of NAFLD among T2DM patients, as we did not observe significant association between them and NAFLD. The same results were reported in previous studies [27,28]. On the other hand, a trend of increased HbA1c level among NAFLD was observed among NAFLD diabetic patients compared with non-NAFLD patients in other studies [10,11]. The cause of this controversy could be explained by the fluctuation in the level of HbA1c that might occur from time to time.

The difference in the prevalence rate of NAFLD and the observed associated factors in the present study compared to a Saudi Study conducted in Jazan region [8] can be explained by geographical differences. Jazan is a coastal area situated at sea level, while Abha is at high altitude (about 2600 m above sea level). Both communities are different in terms of culture and life style. Furthermore, the present study is done at a primary health care level, while the Jazan study is hospital based.

This is the first study to report the prevalence and factors associated with NAFLD among T2DM patients in Abha city, southwestern Saudi Arabia. However, some limitations exist. Firstly, NAFLD cases were screened using conventional ultrasound apparatuses, and were not confirmed by more sophisticated methods. Also, generalization of our results should be performed with some caution, as the study included only T2DM regularly attending PHCCs in Abha city.

## 5. Conclusion

NAFLD is a common association of T2DM. The high prevalence of NAFLD observed in the current study demonstrates an alarming public health problem. Increasing BMI, lower HDL levels, and poor dietary control are significant factors associated with higher risk of NAFLD among T2DM patients.

To minimize the burden of NAFLD among T2DM patients, the following measures are recommended. First, health education should be undertaken for all diabetic patients to improve dietary control and avoid excessive weight gain. Second, NAFLD should be examined among diabetic patients, especially those with abnormal BMI and HDL, for the early detection and proper control of the disease. Thirdly, optimal levels of HDL should be ensured for all diabetic patients.

## Figures and Tables

**Table 1:** Personal and clinical factors associated with NAFLD among the study sample of T2DM patients (*n* = 245).

Factors		NAFLD		cOR (95% CI)	aOR (95% CI)
		No no (%)	Yes no (%)		
Gender	Male	45 (27.8)	117 (72.2)	Ref	Ref
	Female	22 (26.5)	61 (73.5)	1.066 (0.587–1.936)	0.882 (0.391–1.990)
Age (years)	<40	9 (31.0)	20 (69.0)	Ref	Ref
	40–60	24 (22.6)	82 (77.4)	1.537 (0.620–3.815)	1.075 (0.258–4.484)
	≥60	34 (30.9)	76 (69.1)	1.006 (0.415–2.436)	0.885 (0.216–3.625)
BMI (kg/m^2^)	18.5–24.9	12 (63.2)	7 (36.8)	Ref	Ref
	25–29.9	34 (26.8)	93 (73.2)	**4.689 (1.705–12.894)**	**6.112 (1.529–4.432)**
	≥30	21 (21.2)	78 (78.8)	**6.367 (2.230–18.183)**	**10.455 (2.645–41.326)**
Hypertension	No	34 (25.8)	98 (74.2)	Ref	Ref
	yes	33 (29.2)	80 (70.8)	0.841 (0.479–1.476)	0.6229 (0.261–1.485)

NAFLD: Non-alcoholic fatty liver disease; BMI: Body Mass Index; T2DM: Type-2 diabetes Mellitus; cOR: crude Odds Ratio; aOR: adjusted Odds Ratio; 95% CI: 95% Confidence Interval; Ref: reference category; Bold OR: Significant.

**Table 2:** Biochemical Factors associated with NAFLD among the study sample of T2DM patients (*n* = 245).

Factors		NAFLD		cOR (95% CI)	aOR (95% CI)
		No no (%)	Yesno (%)		
HbA1c (%)	<6.5	8 (27.6)	21 (72.4)	Ref	Ref
	6.6-7.5	15 (23.1)	50 (76.9)	1.270 (0.468–3.445)	2.559 (0.661–9.911)
	>7.5	44 (29.1)	107 (70.9)	0.926 (0.382–2.249)	1.004 (0.282–3.569)
Cholesterol (mg/dL)	<200	54 (26.1)	153 (73.9)	Ref	Ref
	≥200	13 (34.2)	25 (65.8)	0.679 (0.324–1.420)	0.452 (0.127–1.616)
Triglyceride (mg/dL)	<150	33 (31.1)	73 (68.9)	Ref	Ref
	>150	34 (24.5)	105 (75.5)	1.396 (0.794–2.455)	1.134 (0.540–2.380)
HDL (mg/dL)	Normal *	61 (25.8)	175 (74.2)	Ref	Ref
	High	6 (66.7)	3 (33.3)	**0.174 (0.042–0.718)**	**0.044 (0.005–0.365)**
LDL (mg/dL)	<100	44 (26.7)	121 (73.3)	Ref	Ref
	>100	23 (28.8)	57 (71.3)	0.901 (0.497–1.633)	0.965 (0.382–2.441)
ALT (IU/L)	<12	38 (35.8)	68 (64.2)	Ref	Ref
	>12	26 (19.8)	105 (80.2)	**2.257 (1.258–4.050)**	**2.355 (1.096–5.062)**
AST (IU/L)	<12	3 (23.1)	10 (76.9)	Ref	Ref
	>12	61 (27.2)	163 (72.8)	0.802 (0.213–3.011)	0.368 (0.074–1.824)

NAFLD: Non-alcoholic fatty liver disease; T2DM: Type-2 diabetes Mellitus; HbA1c: glycosylated hemoglobin; HDL: High Density Lipoprotein; LDL: Low density lipoprotein; ALT: alanine aminotransferase; AST: aspartate aminotransferase; cOR: crude Odds Ratio; aOR: adjusted Odds Ratio; 95% CI: 95% Confidence Interval; Normal * HDL: <40 mg/dL in males and <50 mg/dL for females; Bold OR: Significant.

**Table 3:** Diabetes duration and compliance factors associated with NAFLD among the study sample of T2DM patients (*n* = 245).

Factors		NAFLD		cOR (95% CI)	aOR (95% CI)
		No no (%)	Yes no (%)		
T2DM duration (years)	<5	16 (19.3)	67 (80.7)	Ref	Ref
	5–10	15 (29.4)	36 (70.6)	0.573 (0.254–1.292)	0.625 (0.209–1.866)
	10–15	12 (30.0)	28 (70.0)	0.557 (0.234–1.328)	0.611 (0.179–2.079)
	>15	24 (33.8)	47 (66.2)	**0.468 (0.224–0.975)**	0.432 (0.147–1.268)
Follow up Compliance	Adequate	53 (26.6)	146 (73.4)	Ref	Ref
	Moderate	14 (36.8)	24 (63.2)	0.622 (0.300–1.292)	0.549 (0.201–1.496)
	Poor	0	8 (100.0)	-	-
Diet Compliance	Adequate	26 (44.1)	33 (55.9)	Ref	Ref
	Moderate	33 (28.2)	84 (71.8)	**2.006 (1.044–3.853)**	**2.413 (1.003–5.805)**
	Poor	8 (11.6)	61 (88.4)	**6.008 (2.446–14.754)**	**6.565 (2.056–20.967)**
Drug Compliance	Adequate	56(28.1)	143 (71.9)	Ref	Ref
	Moderate	9 (25.0)	27 (75.0)	1.175 (0.520–2.655)	0.955 (0.335–2.724)
	Poor	2 (20.0)	8 (80.0)	1.566 (0.323–7.605)	0.318 (0.037–2.767)
Exercise Compliance	Adequate	19 (31.7)	41 (68.3)	Ref	Ref
	Moderate	28 (28.0)	72 (72.0)	1.192 (0.593–2.394)	0.737 (0.280–1.943)
	Poor	20 (23.5)	65 (76.5)	1.506 (0.719–3.156)	0.784 (0.278–2.211)

NAFLD: Non-alcoholic fatty liver disease, T2DM: Type-2 diabetes Mellitus, cOR: crude Odds Ratio, aOR: adjusted Odds Ratio, 95% CI: 95% Confidence Interval, Bold OR: Significant.
